# A Type I and Type II microsatellite linkage map of Rainbow trout (*Oncorhynchus mykiss*) with presumptive coverage of all chromosome arms

**DOI:** 10.1186/1471-2164-7-302

**Published:** 2006-11-30

**Authors:** René Guyomard, Stéphane Mauger, Kamila Tabet-Canale, Sylvain Martineau, Carine Genet, Francine Krieg, Edwige Quillet

**Affiliations:** 1 Institut National de la Recherche Agronomique, Laboratoire de Génétique des Poissons, Domaine de Vilvert, Jouy-en-Josas, 78352, France

## Abstract

**Background:**

The development of large genomic resources has become a prerequisite to elucidate the wide-scale evolution of genomes and the molecular basis of complex traits. Linkage maps represent a first level of integration and utilization of such resources and the primary framework for molecular analyses of quantitative traits. Previously published linkage maps have already outlined the main peculiarities of the rainbow trout meiosis and a correspondance between linkage groups and chromosome arms has been recently established using fluorescent *in situ *hybridization. The number of chromosome arms which were covered by these maps remained unknown.

**Results:**

We report an updated linkage map based on segregation analysis of more than nine hundred microsatellite markers in two doubled haploid gynogenetic lines. These markers segregated into 31 linkage groups spanning an approximate total map length of 2750 cM. Centromeres were mapped for all the linkage groups using meiogenetic lines. For each of the 31 linkage groups, the meta or acrocentric structure infered from centromere mapping was identical with those recently found with fluorescent *in situ *hybridization results. The present map is therefore assumed to cover the 52 chromosome arms which constitute the rainbow trout karyotype. Our data confirm the occurrence of a high interference level in this species. Homeologous regions were identified in eleven linkage groups, reflecting the tetraploid nature of the salmonid genome. The data supported the assumption that gene orders are conserved between duplicated groups and that each group is located on a single chromosome arm. Overall, a high congruence with already published rainbow trout linkage maps was found for both gene syntenies and orders.

**Conclusion:**

This new map is likely to cover the whole set of chromosome arms and should provide a useful framework to integrate existing or forthcoming rainbow trout linkage maps and other genomic resources. Since very large numbers of EST containing microsatellite sequences are available in databases, it becomes feasible to construct high-density linkage maps localizing known genes. This will facilitate comparative mapping and, eventually, identification of candidate genes in QTL studies.

## Background

The development of genomic resources is a key step towards further investigation of wide-scale genome evolution, identification and study of gene or gene networks involved in complex characters. Such efforts have naturally focussed on genetic model systems and species of applied biomedical or agronomical importance. In the case of rainbow trout, genetic resources have been primarily developed in order to study the gene networks controlling the phenotype of traits that affect breeding performances or fisheries sustainability and to identify mutations responsible for the genetic variation of these traits. However, the rainbow trout is also a suitable model to investigate more academic scientific questions (reviewed in [1]), such as the evolution of duplicated genomes. Two genome duplications seem to have taken place during the early evolution of vertebrates. Studies based on Hox clusters in several ray-finned fishes and recent comparative analyses of Takifugu and Tetraodon genomes with the Human genome have supported the view that a third whole genome duplication occurred in the ray-finned fish lineage approximately 350 My ago [2-4]. The salmonid family has undergone an additional fourth genome duplication [5,6] which occurred 25–100 My ago. Therefore, the salmonids offer the opportunity to study evolution of duplicated genomes at much earlier stages after a polyploidisation event.

The construction of linkage maps is a primary step towards the description and understanding of the structure and evolution of whole genomes. In rainbow trout, several linkage maps based on segregations at AFLP and microsatellite loci have been already published [7-11] and successfully used to map QTL for growth, disease susceptibility, thermal tolerance, early development [12-16] or to study the duplicated status and chromosomal localisation of gene families playing critical roles in major functions [17-20].

These linkage maps have allowed to ascertain several important features of the meiosis and genome structure in the rainbow trout. Briefly, female maps are three to four times longer than male ones on the average, while telomeric regions show higher average recombination rate in male than in female [10]. Several homeologous regions sharing homology due to the recent tetraploidisation event were identified. Some of these homeologous regions exhibited pseudolinkage [10]. Pseudolinkage refers to a non-random segregation of homeologous regions which results from preferential pairing and alternate disjunction of homeologous regions inherited from the same grand parent. It occurs in males only [21].

The current consensus linkage map consists of 31 major linkage group [8,9]. Since the diploid chromosome number in the rainbow trout ranges from 58 to 64 chromosomes due to a wide range of Robertsonian polymorphism [22], this number falls within the range of expected linkage group numbers. More recently, the rainbow trout genetic linkage groups have been assigned to specific chromosomes using fluorescent in situ hybridisation of BACs [23].

In this article, we report a new rainbow trout map. The main objective was to cover all the chromosomes arms. This information is of importance in rainbow trout since, due to Robertsonian polymorphism, the number of chromosomes and, therefore, of independent linkage groups can vary among families while the number of chromosomes arms are the constant basic characteristic of the rainbow trout karyotype. The construction of the map was based on segregation analysis of nearly one thousand microsatellite markers in two doubled haploid lines, which are currently used in our laboratory. A particular effort was made to map the centromere of each linkage group using meiogynogenetic progeny in order to determine the acrocentric or metacentric structure of each linkage group.

Finally, since approximately one third of the mapped microsatellites originated from ESTs sequences, we attempted to recover conserved syntenies between rainbow trout and zebrafish by comparing the map localisation of homologous sequences in the two species.

## Results

### Number of informative loci

Out of 1321 microsatellite primer pairs tested, 168 did not give any amplification at two annealing temperatures (58 and 52°C) and 366 were monomorphic in the test sample and 796 were informative in one family at least. Three hundred and eighty-nine microsatellite primer pairs were designed from EST sequences. Eighty primer pairs originated from USDA EST sequences, of which eight have been already mapped [7]. The annealing temperature was 58°C for all the informative markers. The 309 markers developed from the SIGENAE-EST database were numbered Omy1009INRA to Omy1513INRA. All these markers including sequence, primer sequences, and PCR conditions were submitted to the STS database in GenBank and assigned accession numbers BV681317 through BV681637. One hundred and five primer pairs amplified duplicated loci. The two female founders of INRA1 and INRA2 were heterozygous for one SNP at BX087958 and only the female parent of INRA1 at BX087759. A total number of 903 loci, including the two SNPs, were informative for the segregation analysis.

### Number of linkage groups

**Figure 1:**
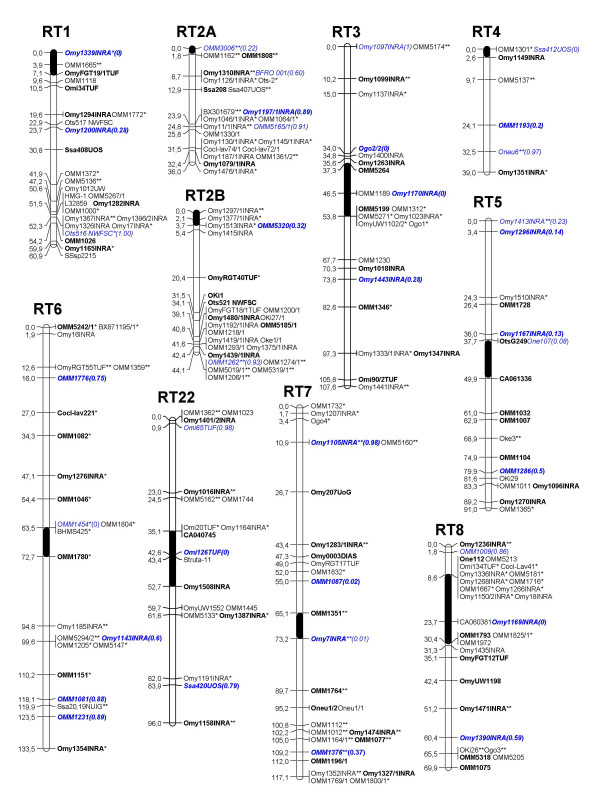
Graphic representation of linkage groups RT1 to RT9. Groups are numbered as in Nichols et al. (2003). Map distances are given in centiMorgan using recombination percentage between loci. Loci with names in blue italic type were used for centromere mapping in the meiogenetic line. Recombination fraction for these loci are given. Putative interval of centromere localisations are materialized by black areas. Loci in bold type are those of the framework map.*: marker informative in INRA1 only; **: marker informative in INRA2 only.

**Figure 2:**
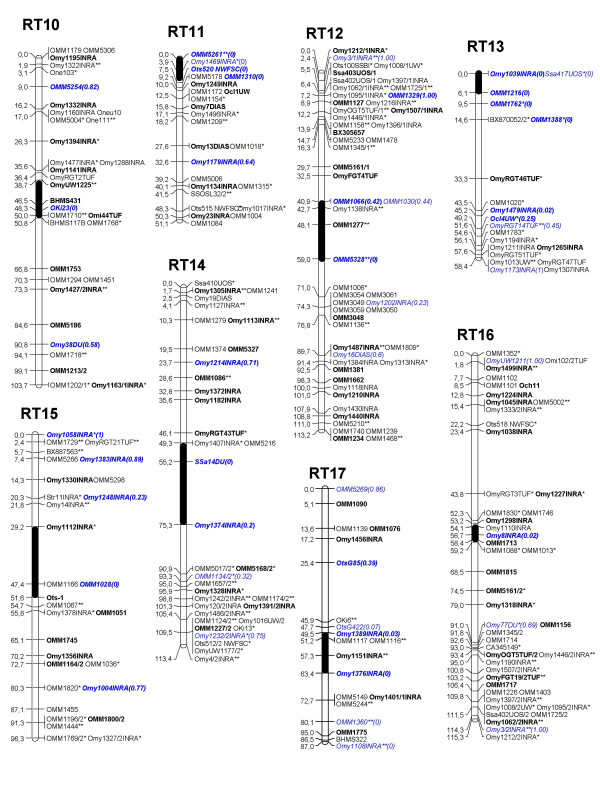
Graphic representation of linkage groups RT10 to RT17. Legends are the same as in Figure 1.

**Figure 3:**
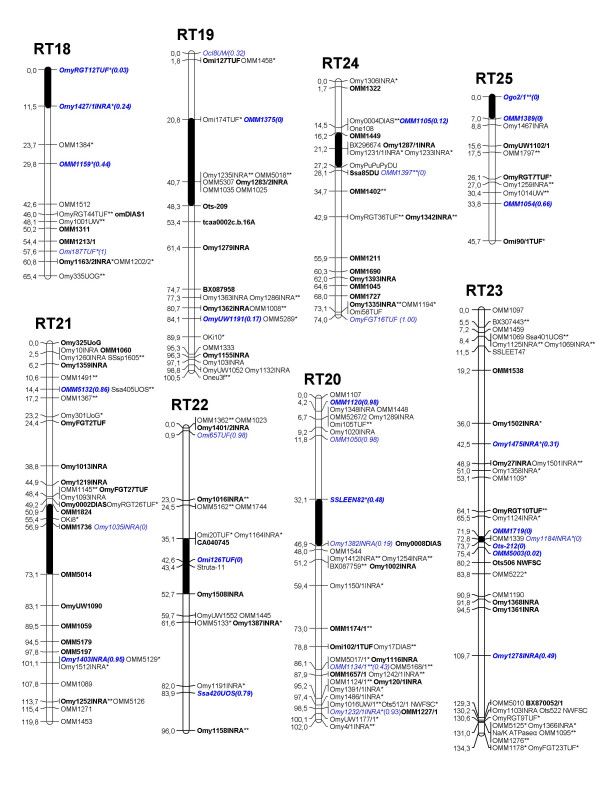
Graphic representation of linkage groups RT18 to RT25. Legends are the same as in Figure 1.

**Figure 4:**
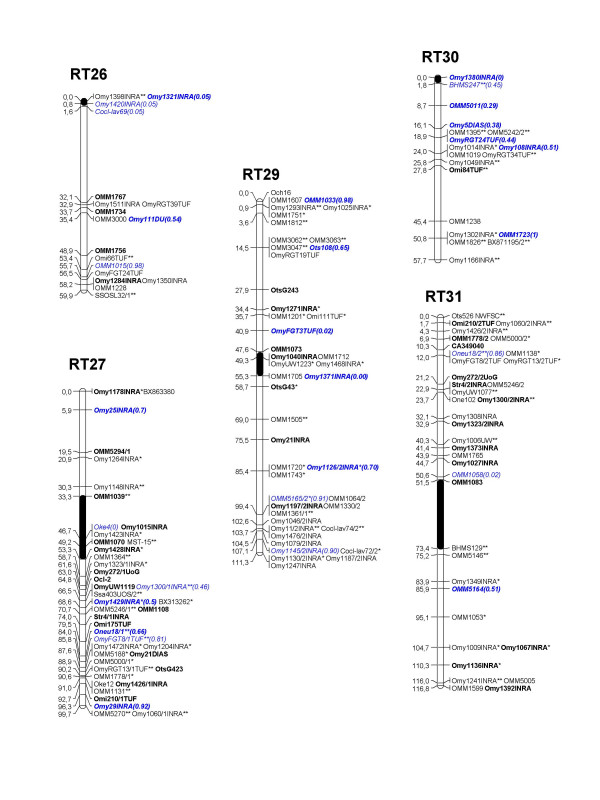
Graphic representation of linkage groups RT26 to RT31 (RT28 does not exist). Legends are the same as in Figure 1.

The comprehensive composite map was obtained by merging the data sets from the two DH families (see Figures 1, 2, 3, 4). This map consisted of 31 linkage groups and one unassigned marker. Correspondences with previous linkage map [8,9] and with specific chromosomes based on FISH results [23] are given (see Additional file 1). RT28 identified in [8] was merged with RT8 here (two unduplicated loci in common). In this study, linkage group RT2 consisted of two unlinked fragments, RT2A and RT2B. Family specific maps consisted of 32 and 35 linkage groups in INRA1 and INRA2 respectively. Four linkage groups (RT6, RT8, RT15 and RT20) consisted of two unlinked fragments in family INRA1 and only one in family INRA2. Similarly, two unlinked fragments were found in family INRA2 for linkage group RT7 instead of one in INRA1. As expected, no significant pseudolinkage was found in female meiosis.

### Centromere mapping and arm numbers of linkage groups

Two to 9 loci per linkage group were mapped in the meiogenetic line (see Figures 1, 2, 3, 4). In all cases, the marker orders obtained for a minimal number of recombination events were identical to the ones found in the two mapping families. For twenty linkage groups (RT3, RT5 to RT10, RT12, RT14 to RT16, RT19 to RT24, RT27, RT29 and RT31; see Figures 1, 2, 3, 4), the loci showing less recombination were found within the linkage group and these groups were assumed to have a metacentric structure. These groups exhibited the highest numbers of double crossovers and almost all of the triple C.O. and quadruple C.O. events found in INRA1 and INRA2 families (see Additional file 1). Double crossover numbers ranged from 13 in RT24 to 38 in RT31 while the other linkage groups had 3 double crossovers or less, with the exception of linkage group RT17 (see below). Altogether, these figures were in agreement with the hypothesis of high interference generally assumed in salmonids and the occurrence of one chiasma per arm in most cases at meiosis I [24,25]. Under this hypothesis, metacentric chromosomes are expected to show mostly zero, one or two crossovers while acrocentric ones mostly zero or one. For each metacentric group, the distribution of the position of the recombination events across all the individuals of INRA1 and INRA2 allowed to determine the positions which minimized the number of double recombinants on each arm. These position delimited putative centromere intervals which were consistent with those of the half-tetrad analysis performed in meiogenetics and sometimes suggested a more precise localisation of centromeres (see Figures 1, 2, 3, 4). These 20 metacentric groups allowed to identify 40 chromosome arms, out of 52. The segregation data in the meiogenetic progeny resulted in the localisation of the centromere at one extremity for the remaining linkage groups and led to the identification of only 11 arms instead of 12. Since RT2A, RT2B, on one hand and RT4 and RT25, were tightly linked and formed metacentric groups in the meiogenetic family, these four linkage groups were likely to be acrocentric in INRA1 and 2. RT1 which corresponds to the chromosome bearing the Sex determinant [8,9] is also stated to be acrocentric or subtelocentric [22]. Among the six remaining linkage groups, RT17 which showed a relatively large number of double crossovers in both INRA1 and 2 families (see Additional file 1) and which was 87 cM in length, emerged as a possible metacentric group.

### Duplicated regions

The 105 duplicated markers were distributed over a total of 27 linkage groups (Table 1). Four linkage groups did not show any duplicated loci and eleven linkage groups shared homologies with two or more different linkage groups (up to four). Twenty-two putative homologous segments were found. Ten of these segment homologies were based on co-localisation of multiple duplicates. One linkage group, RT9, shared homologies with RT2B and RT24 while RT2A shared homology with RT29. The localization of the centromeres was compatible with the assumption that each duplicated region was located on a single chromosome arm (see Figures 1, 2, 3, 4) with the exception of RT3/RT25. These two groups shared three duplicated loci which encompassed the centromeric region of the metacentric RT3 linkage group. Marker orders and orientation with respect to the centromere were conserved between duplicated regions, provided that modifications in the order of very few markers were introduced in the comprehensive map.

### Map length and genome coverage

The map length estimated by the sum of recombination percentage between adjacent markers was 2750 cM and was slightly lower than the value obtained with the Kosambi distance as mapping function (2773 cM). This small difference probably reflected the fact that very few multiple crossovers per arm were detected despite the rather large average number of markers per group (see Additional file 1). Assuming that the centromere positions lied within the intervals proposed (see Figures 1, 2, 3, 4), the total number of multiple C.O. events per arm observed in these two families was estimated to 79 only for a total number of 2975 recombination events. In the meiogenetic family, all genotypes leading to double recombinants per arm were re-genotyped. Only six cases of double crossovers per arm out of a total observed number of 2041 crossovers were found. Double crossovers per arm were not found in a majority of linkage groups even over long map distances since up to 100 % heterozygous progenies were found at distal loci. These finding also supported the hypothesis of high to near-complete interference.

### Comparative mapping with zebrafish

The comparison of 369 rainbow trout EST to the transcript sequences of the zebrafish with BLASTn resulted in 49 hits with e-values < 10^-5^. The assignment of the corresponding loci to the linkage groups of each species and the conserved syntenies are reported in Table 2. These loci covered 21 linkage groups in zebrafish and ten of them housed two or more loci. In all cases, zebrafish linkage groups showed putative homologies with more than one rainbow trout linkage group, irrespective of the duplication in this species. Six syntenies were conserved. In both species, two of them involved two tightly linked loci BX296674/Omy1233INRA and Omy1069INRA/Omy1125INRA) while the four other ones emcompassed large gene distances. It is noteworthy that RT9 and RT2B appeared as a mosaic of non adjacent fragments of the Zebrafish linkage group 3 and that, in zebrafish linkage group 19, the synteny with RT3 was interrupted by a synteny with RT23 (Table 2). A tentative functional annotation of the EST-based microsatellites localised on the map was performed (see Additional file 2). Strong to weak similarities with identified genes were found for 126 of them.

## Discussion

### Number of chromosome arms and genome coverage

The primary objective of this work was to obtain a map covering all the chromosomes arms of the rainbow trout karyotype. The map reported here was based on more than 900 markers and consisted of 31 linkage groups. Analyses of segregation data in both and DH families allowed us to recognize 21 metacentric (with the inclusion of RT17) and ten acrocentric groups. It is noteworthy that our classification was fully supported by a recent Fluorescent *In Situ *Hybridization study [23] which reported the same acro or metacentric structure for each linkage group in rainbow trout (see Additional file 1). Therefore, with respect to our results and the conclusion of the FISH experiments, it can be reasonably assumed that our map covers the 52 chromosome arms which constitute the rainbow trout karyotype.

Our results, both in the mapping families and in the meiogenetic line, were highly consistent with the finding of high interference in salmonid meiosis ([24,25]). Near-complete interference would be the more likely explanation for the absence of multiple recombinants per arm for some linkage groups and the 100% heterozygous progenies for some loci in the meiogenetic family [24,25]. If complete interference is assumed, the length of a linkage group can be estimated by the percentage of recombinants. In most cases, linkage group lengths were close to the expected ones under a complete interference model, i.e. 50 cM for acrocentric groups and 100 cM for metacentric ones in one family at least. Two groups, namely RT2A, RT4 and one arm on RT8, RT17 and RT24, were substantially shorter than 50 cM. The coverage of these groups could be readily improved by integration of additional markers from the microsatellite already published [8,9] or forthcoming linkage maps. Overall, the total map length reported here was rather close to the expected rainbow trout genome length of 2600 cM under complete interference (52 arms × 50 cM). This suggests that the present linkage map provides a good coverage of the rainbow trout genome.

Four linkage groups in INRA1 (RT6, RT8, RT15 and RT20) and one in INRA2 (RT7) were composed of two unlinked fragment. This could reflect a Robertsonian polymorphism or, alternatively, an insufficient local coverage of the linkage group in one family. In the case of a Robertsonian polymorphism, the two unlinked fragments would reflect the independence of the two acrocentric arms. When considering two independent acrocentric chromosomes as a single metacentric one, 1/8 of the progeny is expected to exhibit triple crossovers under complete interference: one crossover on each acrocentric group and one recombinant due to the co-segregation of centromeres from each grandparent. Since we did not find any convincing case of triple crossover at these linkage groups, Robertsonian polymorphism is unlikely to explain the occurrence of unlinked fragments in one of the family. Note also that only two triple crossovers were found instead of 15 (1/8 × 120 individuals) when RT2A and RT2B are grouped, suggesting that these two fragments could be linked in our DH families as in [8,9,23]

Assignment of each linkage group to a specific chromosome[23] will allow to replace the current linkage group numbering by a nomenclature based on chromosome size as in other species. The adoption of a definitive nomenclature will require a clear identification of all the chromosome arm associations involved in the Robertsonian polymorphism in rainbow trout

### Alignment with other rainbow trout maps and zebrafish map

A total number of 214 microsatellite loci were common to the female-specific maps published by [8] and this one. Syntenies were not conserved for eight loci which mapped to different linkage groups in each map. None of these inconsistencies could be explained by a misinterpretation of a homeology as a homology since the misassigned loci did not map to homeologous regions. The 206 remaining markers were correctly assigned to a total number of 23 linkage groups. The conservation of gene orders was checked for 21 linkage groups which shared more than two markers in the two maps. In most cases, inconsistencies in gene orders resulted from the unstable position of one marker only or involved markers separated by short distances. Only RT15 and RT27 showed important discrepancies. Despite the fact that four families were genotyped (two in this study and two in [8]), in all cases, discrepancies in gene orders were detected in only two families, the two others being uninformative with respect to the order of the problematic markers. The correct order in these groups remains to be validated. The two maps were also congruent for the localisation of the centromeres. All the linkage groups which showed a centromere in central position in the map of [8] were also stated as being metacentric in our map.

One hundred and forteen microsatellite loci covering all the linkage groups were common to the map of [9] and ours. Only nine markers were not assigned to the same group. Two discrepancies resulted from comparison of homeologous loci. [9] map was a male-specific linkage map obtained by genotyping androgenetic doubled haploid progeny. Therefore, most of the microsatellite markers clustered tightly in the centromeric regions as expected in male and this map did not provide sufficient information on gene orders.

Overall, both syntenies and gene order appear to be conserved among the different linkage maps.

The localisation of type I marker on this map has provided some evidences of conservation of syntenies between zebrafish and rainbow trout, though it is not the rule. Of particular interest are the alignment of zebrafish linkage groups 3 and 19 on the linkage map of rainbow trout and the complex architecture of the syntenies found. Even if the comparison of the zebrafish and rainbow trout genome is still very preliminary and based on a crude assessment of sequence homologies, these findings suggest that chromosome rearrangements have been frequent between zebrafish and rainbow trout at least over long map distances, but that cases of conserved syntenies can be found.

### Duplicated regions

Extensive gene duplication has been reported early in salmonids and has been a crucial argument in favour of the hypothesis of tetraploidy in salmonids. Our data confirm several homeology between linkage groups already reported or hypothesized, namely RT2–RT9, RT2–RT29, RT3–RT25, RT7–RT15, RT10–RT18, RT12–RT16, RT14–RT20 and RT27–RT31 ([8-10]). With respect to these previous studies, two new homeologous regions were identified here: RT6–30 and RT9–24. Finally, a new homeology was suggested for RT17 and 22 which shared Omy1401INRA in this study and OmyRGT6TUF in [8]. Our finding that gene orders are conserved between homeologous markers is congruent with segregation data which indicate the occurrence of pseudolinkage between duplicated pairs belonging to homeologous regions. Such pseudolinkages result from pairing between homeologous regions and alternate segregation [21] and it is likely that rearrangement in gene orders would prevent or impede such homeologous pairing. Our results were also compatible with the assumption that each confirmed homeologous region (i.e. defined by two or more shared duplicated loci) mapped to a single chromosome arms, with the exception of the RT3/RT25. These two linkage groups showed a meta and acrocentric structure respectively and the three shared duplicated loci were not found on the same arm of RT3. This could eventually reflect a pericentric inversion involving one marker on RT3, but this exception remains to be validated. When two homologies per arm were found, one of these homologies involved one single pair of duplicated loci. Thus, the occurrence of more than one homology per arm remains hypothetical for the moment. Although the occurrence of more than one strongly conserved homeologous region per arm cannot be excluded, it is likely to lead to rather complex configurations of chromosome pairing and to unbalanced segregations at meiosis.

No unique pattern of localisation of the homeologous segment on chromosome emerged from this study. The homeologous regions sharing the largest number of duplicated loci, namely RT2A/29, RT2B/9, RT12/16, RT14/RT20 and RT27/31 did not include the centromeric region. This is in agreement with the models of tetravalent formation, chromatide exchange and conservation of homology between homeologous distal region of chromosomes proposed by [21] and [5]. In the other cases, homeology could concern whole arms or be restricted to the region proximal to the centromere as for RT9/14. More detailed description of the structure of homeologous linkage groups, with the addition of numerous additional loci, and sequencing of large BAC fragments mapping to these linkage groups, both in duplicated and apparently not duplicated regions, could be a useful step towards the understanding of the evolution of a tetraploidized genome.

## Conclusion

The present work provides a linkage map which is likely to cover most of the genome and to identify all the chromosome arms. It should be very helpful to distinguish between unlinked fragments due to insufficient marker density, Robertsonian polymorphism and pseudolinkage in future studies. In turn, this work should benefit from the other maps under construction, specifically from the information derived from male meiosis. In contrast with female specific maps which show rather uniform distribution of recombination events along arms ([8]; this study), male specific maps exhibit more recombination towards the telomeric ends and could be more resolutive for these regions. Finally, approximately four hundred microsatellite markers developed from EST have been mapped in this study, which is only a very small fraction of this type of markers available in public databases. This provides a means to generate high-density gene maps in rainbow trout and makes feasible comparative mapping with phylogenetically distant model species such as zebrafish (this study; [9]) or tetraodon [7]. Our preliminary data suggest that conservation of syntenies between rainbow trout and model species could occasionally facilitate fine-mapping of QTLs and, eventually, identification of candidate genes.

## Methods

### Mapping resource populations

The linkage map was constructed using two unrelated doubled haploid (DH) lines (60 individuals per line). The source population was an experimental INRA rainbow trout strain. A grand-parental population of all homozygous doubled haploids was first established, using the mitotic gynogenesis [26]. Briefly, ova from individual females were fertilized with UV irradiated sperm, and diploidy was restored by blocking first embryonic cleavage with a heat shock. Some of the DH progenies were sex-reversed by early hormonal treatment in order to obtain functional males. The next generation, DH males and DH females from different families were single-pair mated, producing all female F1 hybrid progeny between homozygous lines. Two of those females were reproduced using a second round of mitotic gynogenesis in order to produce the two mapping DH families used in this work (INRA1 and INRA2).

Centromeres were mapped using a diploid meiogenetic family (60 individuals). To produce this meiogenetic family, eggs from a single female were fertilized with UV irradiated sperm and diploidy was restored by retention of the second polar body [27].

All the individuals (4 DH grand-parents, 3 mothers and progeny) were fin clipped and fin clips were stored in 95% ethanol for subsequent analyses.

### Microsatellite and SNP genotyping

A total number of 1321 primer pairs were tested. Name, references, primer sequences of markers not submitted to GenBank are given (see Additional file 3). Five hundred and seven new primer pairs have been developed from EST sequences containing microsatellite motives. A SIGENAE ENSEMBL Rainbow Trout EST Contig version 3.0 was extracted from the SIGENAE-EST database [28]. This file was screened for short tandem repeats (two to five nucleotides) with a software developed by F. Mougel (unpublished). The lower repeat numbers were fixed to eight for di and tri-nucleotide motives and to five for tetra and penta-nucleotide repeats. Primers were designed with PRIMER3 software [29]. Eight hundred and fourteen already published microsatellite markers were also selected in databases or in publications. To discard redundant markers prior to PCR tests, sequence homologies were first checked by Blast at low stringency (Blastall command in GCG package; chosen E-value: 10^-3^), then manually validated. New microsatellite markers were named in accordance with the convention outlined by [30]. For all microsatellite markers, species abbreviations and microsatellite source acronyms are given in [9]. PCR tests were done on a sample consisting of the two mothers and the four grand-parents of DH progeny. Duplicated loci were named according to the nomenclature described in [31].

Genomic DNA samples were prepared from ethanol preserved fin tissues following simplified phenol extraction and ethanol precipitation procedures [32]. The PCR amplifications, separation and visualization of the PCR products were carried out according to [31], at the annealing temperature of 58°C. When PCR amplification was unsuccessful, the annealing temperature of 52°C was tested. The Mg^++ ^concentration was fixed to 2 mM.

Two sequences, BX087958 and BX087759, were mapped by SNP-based genotyping using sequencing and PCR-RFLP procedures. Fragments of 550 and 650 base pairs respectively were amplified by PCR. Amplifications (30 cycles, 58°C annealing, 1 mn elongation) were carried out in 20 μl reactions containing 40 ng of genomic DNA template, 1X reaction buffer (Promega), 0.4 μM of each primer, 250 μM of each dNTP, 2 mM MgCl2, 0.6 units Taq polymerase (Promega). SNP polymorphism in females was detected by sequencing. PCR products were purified by treatment with 2 units of exonuclease 1 and one unit of shrimp alkaline phosphatase. SNP polymorphism in the mothers of DH progeny was detected by sequencing using the ABI Big Dye terminator chemistry on an ABI 310 sequencer. Progeny were genotyped by PCR-RFLP methods: BX087958 and BX087579 PCR products were cut with BsrB I and Taq I respectively following Biolabs protocol and fragments were separated in 2% agarose.

### Linkage analysis and centromere mapping

Genotypes were converted into a backcross format with linkage phase deduced from the genotypes of the grandparents, each offspring being equivalent to one gamete. Since linkage phase was known, the two data files obtained for each female were merged and analysed as a single data set. Linkage groups, gene orders and map distances were obtained with CARTHAGENE [33]. Linkage groups were generated with Group command for a LOD threshold of 3. For each linkage group, a 1000:1 framework map (likelihood of the map at least 1000-fold higher than the next highest likelihood using the same markers in alternate orders) was also built by using a stepwise locus adding strategy (BuilFW command). When possible, microsatellites from ESTs were preferred to anonymous microsatellites. The provisional frameworks were checked for alternative orders with higher likelihood using Flips, Greedy and Annealing commands which test inversions and permutations of group of markers. Finally, a comprehensive map including all the markers was built, by adding all the remaining markers at the most likely location with Mapcomp command and optimized with Flip command (or details on commands, see Carthagene user guide at [34].

Though it was not expected in female meiosis, the occurrence of pseudo-linkage was checked using a genotype file with inverted linkage phase.

The linkage group numbers followed the current nomenclature [8,9]. Correspondences between maps were based on the occurrence of several markers common to these different maps. The map published by Nichols *et al*. [9] also consisted of ten additional small group which were not taken into account. In addition, linkage group OA-XXVIII was no longer considered in Nichols *et al*. [9].

Graphic representations of linkage groups were generated with MAPCHART version 2.1 [35]. The percentage of recombinants was used as the mapping function to account for the high levels of chiasma interference which are usually reported for rainbow trout [10,24,25]

Centromeres were mapped through genotyping of meiogenetic progeny for several loci per linkage group. For each linkage group, loci were ordered manually in such a way that the number of recombination events was minimum. This condition is expected under the hypothesis of complete interference. The most likely interval in which the centromere lies was deduced from the gene-centromere distances (estimated as half the proportion of heterozygotes in the meiogynogen progeny), avoiding, as far as possible, double recombinants per arm (hypothesis of nearly complete interference). Details of this half-tetrad analysis can be found in [36].

### Comparative mapping with zebrafish, *Danio rerio*

The EST sequences of rainbow trout were masked from known repeats and vector sequences and compared to all the transcript sequences of the zebrafish using the Iccare Web server [37] with the Blastn option of the BLAST program. Only the Highest Scoring Pairs (HSP) with an expected value less than 10^-5 ^were kept. The output file displayed the BLAST results and the location of the rainbow trout genes on the chromosomes of the zebrafish for each query sequence showing significant homology.

## Authors' contributions

RG conceived the project, supervised data analyses and interpretations and wrote the manuscript. SMR contributed to genotyping, collection of segregation data, data treatments and map construction. KTC produced the EST-based microsatellites from the EST-SIGENAE database, contributed to genotyping, segregation data collection and analyses. SMU contributed to the EST-SIGENAE database screening, genotyping, segregation data analyses. CG contributed to data analysis, performed comparative mapping with zebrafish and tentative annotation of rainbow trout sequences and contributed to manuscript drafting. FK contributed to EST-SYGENAE database screening and genotyping. EQ contributed to project conception and manuscript drafting and produced the mapping DH families. All authors read and approved the final manuscript.

## Supplementary Material

Additional file 1 Correspondence of the map reported in this study with already published linkage maps and with chromosome identified by FISH. Number of markers and crossovers per linkage group. Click here for file

Additional file 2 EST accession number or reference for microsatellite loci unavailable in GENBANK. Click here for file

Additional file 3 Functional Annotation of microsatellites markers associated to ESTs. Tentative annotations from Oncorhynchus mykiss UniGene Build [38] or from rainbow trout gene index version 5.0 [39] were retrieved with the Rainbow Trout EST Sigenae/Ensembl EST contig browser. Markers are annotated with NCBI protein accession number [40] for Unigene annotation or from Uniprot accession number [41] for TIGR annotation. Unigene contig name, Unigene contig description, TIGR contig and TIGR contig description are given. Click here for file

## Figures and Tables

**Table 1:** Oxford grid showing the homeologous relationships between linkage groups in rainbow trout. Linkage group numbers are indicated on first line and row. Number of markers shared between duplicated regions are given

	**1**	**2**	**3**	**4**	**5**	**6**	**7**	**8**	**9**	**10**	**11**	**12**	**13**	**14**	**15**	**16**	**17**	**18**	**19**	**20**	**21**	**22**	**23**	**24**	**25**	**26**	**27**	**29**	**30**	**31**
**1**												1				1				1										
**2**									19																			15		
**3**																1									3					
**4**																														
**5**																														
**6**																											1		2	
**7**															5				1											
**8**									1											1										
**9**																								2						
**10**																		4												
**11**																										1				
**12**																14											1			
**13**																							1							
**14**																				16										
**15**																														
**16**																				1										
**17**																						1								
**18**																														
**19**																														
**20**																														
**21**																														
**22**																														
**23**																														
**24**																														
**25**																														
**26**																														
**27**																														13
**29**																														
**30**																														
**31**																														

**Table 2:** Linkage group (LG) assignations are given for both species. In rainbow trout, position on the linkage group are given starting from the presumably closest marker to the centromere

EST	Rainbow trout		Zebrafish	
	
	LG	Position on LG	LG	Position on LG
Omy1017INRA	11	48.3	1	53.2
Omy1212INRA	12/16	40.9/58.6	1	29.2
Omy1242INRA	14/20	43.6/39.9	1	28.0
Omy1219INRA	21	0.11	1	42.8
OMM5188	27	40.9	1	47.1
BX305657	12	27.0	2	35.7
Omy1298INRA	16A	3.5	2	1.9
CA345149	16B	36.3	2	17.6
BX870052	23	56.5	2	34.3
Omy1192INRA	9A/2B	40.8/48.1	3	2.8
BX296674	24/9B	6.0	3	7.9
Omy1233INRA	24/9B	6.0	3	8.6
OMM5127	9A	3.4	3	8.6
OMM5019	2B/9A	44.1/48.1	3	14.7
OMM5000	27B/31B	42.2/43.7	3	21.5
Omy1261INRA	9A	0.0	3	28.6
Omy1389INRA	17	0.0	3	44.1
Omy1474INRA	7	50.0	4	7.6
Omy1202INRA	12B	33.4	5	41.0
BX871195	6/30	63.5/58.8	6	23.1
Omy1160INRA	10A	31.3	8	36.0
Omy1510INRA	5	13.4	9	26.7
OMM5205	8	31.3	10	2.1
Omy1002INRA	20	4.3	10	2.3
Omy1512INRA	21	44.2	10	24.0
Omy1321INRA	26	0.0	10	29.7
Omy1271INRA	29	6.5	11	1.2
BX887563	15	41.7	12	23.0
OMM5244	17	17.2	12	20.9
Omy1049INRA	30	25.8	12	31.6
Omy1130INRA	2A/29B	31.5	13	44.2
Omy1358INRA	23	21.8	13	34.7
OMM5264	3A	9.2	14	18.0
Omy1300INRA	27/31	19.8/26.9	16	42.9
BX307443	23	67.3	17	12.9
OMM5137	4	9.7	18	39.3
OMM5199	3B	7.3	19	0.8
Na/K ATPaseα	23B	58.2	19	1.8
Omy1278INRA	23B	36.9	19	20.9
Omy1441INRA	3B	61.1	19	62.4
Omy1227INRA	16A	12.9	19	68.0
Omy1112INRA	15	18.2	20	6.6
Omy1069INRA	23A	64.4	20	34.0
Omy1125INRA	23A	64.4	20	37.5
OMM5006	11	39.2	23	52.1
OMM5002	16A	41.3	23	52.1
Omy1260INRA	21	54.4	23	31.2
Omy1286INRA	19	56.5	25	10.9
